# The Tumor–Fat Interface Volume of Breast Cancer on Pretreatment MRI Is Associated with a Pathologic Response to Neoadjuvant Chemotherapy

**DOI:** 10.3390/biology9110391

**Published:** 2020-11-10

**Authors:** Hwan-ho Cho, Minsu Park, Hyunjin Park, Eun Sook Ko, Na Young Hwang, Young-Hyuck Im, Kyounglan Ko, Sung Hoon Sim

**Affiliations:** 1 Department of Electronic, Electrical and Computer Engineering, Sungkyunkwan University, 2066 Seobu-ro, Jangan-gu, Suwon 16419, Korea; guraud0810@skku.edu; 2 Center for Neuroscience Imaging Research, Institute for Basic Science (IBS), Sungkyunkwan University, 2066 Seobu-ro, Jangan-gu, Suwon 16419, Korea; 3 Department of Statistics, Keimyung University, 1095 Dalgubeol-daero, Dalseo-gu, Daegu 42601, Korea; minsu.park51@gmail.com; 4 School of Electronic and Electrical Engineering, Sungkyunkwan University, 2066 Seobu-ro, Jangan-gu, Suwon 16419, Korea; 5 Department of Radiology, Samsung Medical Center, Sungkyunkwan University School of Medicine, 81 Irwon-ro, Gangnam-gu, Seoul 06351, Korea; 6 Statistics and Data Center, Research Institute for Future Medicine, Samsung Medical Center, Seoul 06351, Korea; ny.hwang@sbri.co.kr; 7 Division of Hematology/Oncology, Department of Medicine, Samsung Medical Center, Sungkyunkwan University School of Medicine, 81 Irwon-ro, Gangnam-gu, Seoul 06351, Korea; imyh00@skku.edu; 8 Department of Radiology & Cancer Research Institute, National Cancer Center, Goyang-si 10408, Korea; kokr@ncc.re.kr; 9 Division of Hematology/Oncology, Department of Medicine, National Cancer Center, Goyang-si 10408, Korea; simsh@ncc.re.kr

**Keywords:** breast cancer, neoadjuvant chemotherapy, MRI, adipose tissue, pathological complete response

## Abstract

**Simple Summary:**

Contact between a tumor and the adjacent fat is a potential biomarker to predict the therapy response in breast cancer, but it has not been quantitatively explored. In this study, we measured the direct contact between the tumor and adjacent fat using breast magnetic resonance imaging with machine learning and found that patients with a greater volume of contact between tumor and fat were less likely to have a complete pathological response. Our results suggest that the volume of the tumor–fat interface is a potential prognostic imaging biomarker to predict the treatment response to neoadjuvant chemotherapy.

**Abstract:**

Adipocytes are active sources of numerous adipokines that work in both a paracrine and endocrine manner. It is not known that the direct contact between tumor and neighboring fat measured by pretreatment breast magnetic resonance imaging (MRI) affects treatment outcomes to neoadjuvant chemotherapy (NAC) in breast cancer patients. A biomarker quantifying the tumor–fat interface volume from pretreatment MRI was proposed and used to predict pathologic complete response (pCR) in breast cancer patients treated with NAC. The tumor–fat interface volume was computed with data-driven clustering using multiphasic MRI. Our approach was developed and validated in two cohorts consisting of 1140 patients. A high tumor–fat interface volume was significantly associated with a non-pCR in both the development and validation cohorts (*p* = 0.030 and *p* = 0.037, respectively). Quantitative measurement of the tumor–fat interface volume based on pretreatment MRI may be useful for precision medicine and subsequently influence the treatment strategy of patients.

## 1. Introduction

Neoadjuvant chemotherapy (NAC) has become the standard care for patients with locally advanced breast cancer and those with high-risk early breast cancer [1,2,3]. Many studies have reported that a pathologic complete response (pCR) can be used as a surrogate endpoint for the prediction of long-term clinical benefits, such as disease-free survival and overall survival [4,5]. One of the greatest merits of NAC would be to provide an in vivo assessment of tumor response. This information could allow clinicians to change regimens based on knowledge gained from NAC [6,7].

Adipose tissue mainly consists of mature adipocytes. The systemic effects of obesity on cancer mainly relate to the consequences of adipocyte dysfunction [8]. Studying the role of adipocytes in cancer occurrence or progression has been actively performed due to the established link between obesity and cancer. It has been known that adipocytes are not only energy reservoirs but also active sources of numerous adipokines, including leptin, adiponectin, and tumor necrosis factor (TNF)-α, that work in both a paracrine and endocrine manner [9,10]. These adipokines, acting locally or systematically, could play important roles in the growth, local invasion, metastasis, and resistance to treatment of different types of cancer [11]. Recent studies have suggested that adipocytes promote cancer resistance to chemotherapy and radiotherapy via adipokines or adipocyte-induced hypoxia [12,13,14]. Several studies have demonstrated that breast cancer cells and neighboring adipocytes of the tumor microenvironment also interact with each other directly to create advantageous inflammatory microenvironments which, in turn, further support tumor progression [15,16]. Although most of the experimental studies performed regarding adipocytes have emphasized their paracrine role, to date, the effects of neighboring adipose tissue and tumor cells on the response to treatment has never been determined using conventional imaging modalities in a NAC setting.

Based on such a background, we hypothesized that breast cancer patients with a higher volume of direct contact between the tumor and neighboring fat, defined as the tumor–fat interface, might have a worse pathologic response to NAC. Therefore, the purpose of this study was to evaluate the clinical significance of the tumor–fat interface volume measured by pretreatment MRI for predicting pCR in breast cancer patients treated with NAC, and to validate the effect of this new quantitative imaging biomarker on pCR using an external cohort.

## 2. Materials and Methods

### 2.1. Patients

This retrospective multicenter study was conducted in accordance with the Declaration of Helsinki and was approved by the Institutional Review Board of each participating hospital (SMC 2019-12-076-001, NCC 2020-0075). The requirement for informed consent was waived.

**Figure 1:**
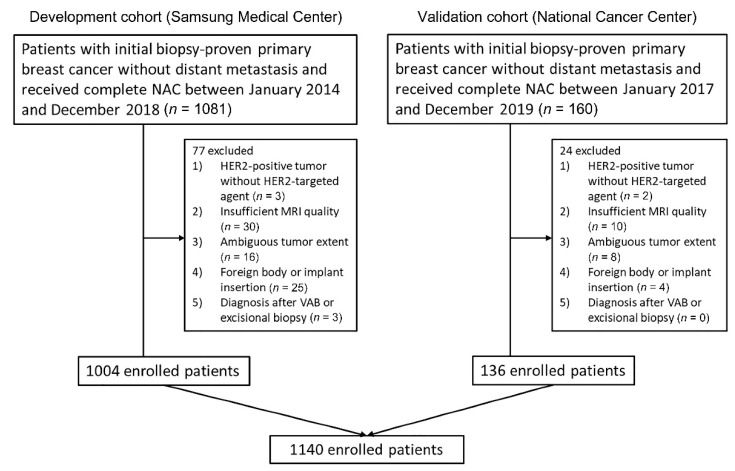
Flow chart of the study population. In total 1140 patients were included according to the inclusion and exclusion criteria from two hospitals.

Our cohort consisted of breast cancer patients treated with NAC from two hospitals. Patients from Samsung Medical Center (SMC) were used as the development cohort and patients from the National Cancer Center (NCC) were used as the validation cohort. The flowchart in Figure 1 depicts an overview of the datasets used in this study.

The inclusion criteria were as follows: (1) patients with initial biopsy-proven unilateral invasive ductal carcinoma (IDC) without distant metastasis; (2) patients who underwent complete NAC with no prior treatment; (3) surgery was performed after the completion of NAC at our institution; and (4) pretreatment breast MRI was conducted within 1 month prior to NAC. The exclusion criteria were as follows: (1) patients with HER2-positive tumors who did not undergo HER2-targeted therapy (*n* = 5); (2) insufficient MRI quality to obtain accurate measurements because of motion artifact or incomplete fat suppression (*n* = 40); (3) ambiguous tumor extent (*n* = 24); (4) foreign body injection or implant insertion for breast augmentation (*n* = 29); and (5) diagnosis after vacuum-assisted biopsy or excisional biopsy (*n* = 3).

Finally, 1140 women (mean age, 47.5 years; age range, 24–75 years) were included in the present study (Figure 1).

The following four NAC regimens were employed: adriamycin with cyclophosphamide (AC); adriamycin with cyclophosphamide plus docetaxel (AC-T); AC-T with trastuzumab (ACTH); or docetaxel, carboplatin, trastuzumab, and pertuzumab (TCHP).

### 2.2. MRI Protocol

Breast MRI for each patient was performed within 1 month of NAC commencement. All breast MRI was performed on a 3-Tesla or 1.5-Tesla scanner (Achieva, Philips Healthcare, Best, The Netherlands). Axial images of both breasts were acquired with the patient in the prone position. All scanners used in this study and the protocols are described in the Supplementary document.

### 2.3. Preparation of MRI for Measurement

Pretreatment MRI data from two participating hospitals were collected for segmentation and quantitative measurements. Whole fat-suppressed dynamic contrast-enhanced T1-weighted images and contrast-enhanced T1-weighted subtraction MR images were retrieved from the Picture Archiving Communication System and loaded onto a workstation for further quantitative measurement. Subtraction images from contrast-enhanced images at 90 s after contrast injection to pre-enhanced images were assessed. Segmentation of the ipsilateral chest wall in the slices of the whole tumor was delineated manually via the MRIcro software on every fifth slice of the contrast-enhanced T1-weighted images. The MR images were reviewed by a radiologist with 13 years of experience in breast MRI (E.S.K.) who was blinded to the pathologic outcome. Segmentation of the two data sets was performed by one radiologist (E.S.K.) for consistency.

To calculate the interobserver agreement of quantitative measurements, we randomly selected 50 patients using statistical software. Two radiologists (E.S.K. and K.R.K.) independently segmented the chest wall on the same contrast-enhanced T1-weighted images.

### 2.4. Quantitative Measurements of Tumor–Fat Interface

To segment the breast region, a search region was set up at the anterior portion of the chest wall. A k-means clustering algorithm was used to separate the fat, tumor, and normal fibroglandular tissue. Full details are provided in the Supplementary document.

**Figure 2:**
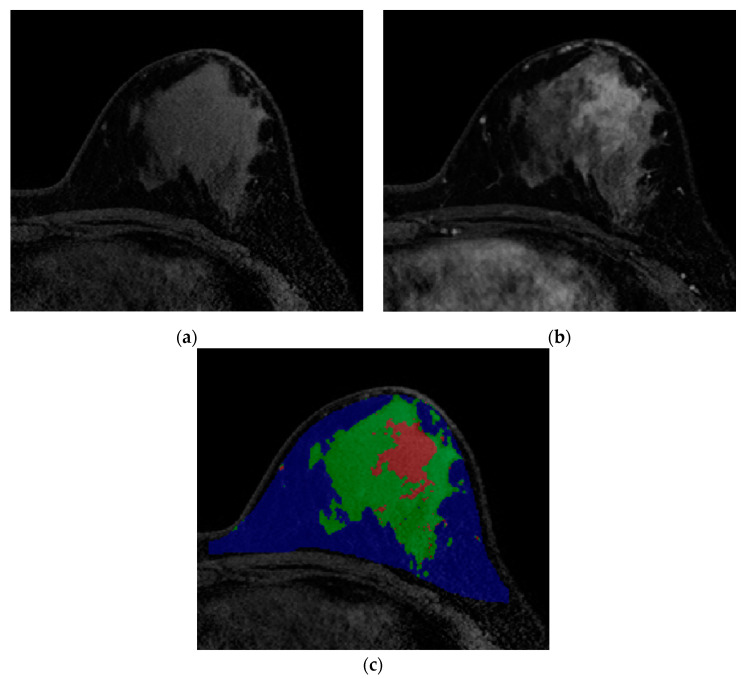
Images from breast MRI of a 42-year-old woman with triple-negative invasive ductal carcinoma in the left breast. (**a**) Pre-enhanced T1-weighted axial image discriminates fat and non-fat regions. (**b**) Contrast-enhanced T1-weighted image shows a 4 cm malignant mass. At this stage, differentiation between tumor and normal fibroglandular tissue is conducted. (**c**) A color-overlay image showing differentiation between fat (blue), normal fibroglandular tissue (green), and tumor (red). In this patient, the tumor-fat interface volume was 1.80 cm^3^ and classified as low interface group. Final pathology after surgery revealed a pCR.

We defined the tumor–fat interface as the direct contacting plane between fat and tumor, which presented mechanically as a collection of fat pixels with neighboring tumor pixels. Within the fat region, if a fat pixel was within one pixel of the tumor pixels in a 6-pixel-adjacency 3D neighborhood, it was deemed as the interface pixel. The procedure was repeated for all pixels in the fat region and we summed all the interface pixels. Through these processes, breast volume (cm^3^), fat volume (cm^3^), tumor volume (cm^3^), normal fibroglandular tissue volume (cm^3^), and tumor–fat interface volume (cm^3^) were quantitatively calculated (Figure 2).

### 2.5. Clinical and Pathological Evaluation

Clinical staging of patients was performed according to the radiological and clinical findings in accordance with the criteria of The American Joint Committee on Cancer (AJCC) 7th edition [17]. Body mass index (BMI) (kg/m^2^) was calculated as weight (kg) divided by square of height (m^2^). Height and weight measured at a clinical visit close to initiation of chemotherapy were used to calculate BMI. Patients were classified into the obese group according to the BMI cutoff (≥25) proposed by World Health Organization for Asian populations [18]. Menopausal status, background parenchymal enhancement (BPE), and mammographic breast density at diagnosis were also recorded.

Final histopathological results of surgical specimens were reviewed to determine the presence of residual invasive or carcinoma in situ components of the tumor in the breast or axilla. The expression status of the estrogen receptor (ER), progesterone receptor (PR), and human epidermal growth factor receptor 2 (HER2) was determined from the immunohistochemical results of core biopsies performed prior to chemotherapy. The cutoff for ER/PR positivity was set at 1% [19]. Hormone receptor (HR) was defined as positive when either ER or PR was expressed. The cutoff for Ki-67 was set at 20%. Tumors with HER2 scores of 3+ were considered positive. In the case of 2+ scores, silver in situ hybridization (SISH) was used to determine HER2 amplification. Breast cancers were divided into four molecular subtypes based on the immunohistochemical or SISH findings for ER, PR, HER2 as follows: HR+/HER2−, HR+/HER2+, HR−/HER2+, and HR−/HER2−.

The pCR was defined as complete disappearance of all invasive tumor cells in the breast and regional lymph nodes regardless of the presence of residual DCIS (ypT0/is N0) [20].

### 2.6. Statistical Analysis

The potential association of the tumor–fat interface volume with the pathologic response after NAC was first assessed in the SMC set (development cohort) and then validated in the NCC set (validation cohort).

Clinical and pathological features were statistically compared between pCR and non-pCR groups using the Fisher’s exact test or the chi-squared test for categorical variables and the Wilcoxon rank sum test for continuous variables. Quantitative values measured from MRI, including the tumor–fat interface volume, were compared between the two groups according to the pathologic response using the Wilcoxon rank sum test. To dichotomize tumor–fat interface volume for analyzing the effect on the pathologic response to NAC, the optimal cutoff points were determined by the Youden’s J index in the development cohort [21]. Patients were divided into the high or the low interface group according to the optimal cutoff point of the tumor–fat interface volume. The characteristics of the patients according to the dichotomized interface group were also compared. Univariable logistic regression analysis was used to analyze the effects of clinicopathological variables and quantitative volumetric factors obtained via MRI. Variables showing a significant association (*p* < 0.05) with the pathologic response in univariable analyses were input variables for the multivariable logistic regression analyses. Variance inflation factor (VIF) values were computed for each predictor in order to check for multicollinearity, and none exceeded 5.

Patient characteristics in the development and validation cohort were compared using the Wilcoxon rank sum test for continuous variables and the chi-squared test or Fisher’s exact test for categorical variables. To validate our results from the development cohort, univariable and multivariable logistic regression analyses were performed to analyze the effects of clinicopathological variables and quantitative volumetric factors obtained via MRI in the validation cohort.

To demonstrate the value of the tumor–fat interface volume on the prediction of pathologic response, the following nested prediction models were compared; the conventional prediction model and combined prediction model. The conventional prediction model composed of independent various risk factors based on multivariable logistic regression analysis. The combined prediction model incorporated the conventional model and the interface group. The performance of the two prediction models, both in the development and validation cohorts, was analyzed using DeLong test for two correlated receiver operating characteristic (ROC) curves, reclassification improvement (NRI), integrated discrimination improvement (IDI), and Akaike information criterion (AIC). To evaluate the correlation between the quantitative measurement values, Spearman’s rank correlation analysis was performed.

Next, to evaluate whether the prognostic value of the tumor–fat interface volume is different according to tumor subtypes, subgroup analysis was performed after adjusting age, BMI, T stage, N stage, ER, PR, HER2, Ki-67, and menopausal status.

A *p*-value < 0.05 in two-sided tests was considered to be statistically significant. All statistical analyses were performed using R Statistical Software (version 3.6.4; R Foundation for Statistical Computing, Vienna, Austria) or SAS software (version 9.4, SAS Institute, Cary, NC, USA).

## 3. Results

### 3.1. Patient Outcomes

Table 1 shows the general characteristics of patients enrolled in the development and validation cohorts according to pathologic response to NAC. The pCR rate was 36.8% and 33.1% in the development and validation cohort, respectively. There was no significant difference in BMI between the pCR and non-pCR groups with regards to both continuous (*p* = 0.817 and *p* = 0.627 for the development and validation cohorts, respectively) and dichotomized variables (*p* = 0.775 and *p* > 0.999 for the development and validation cohorts, respectively). ER-negativity, PR-negativity, and HER2-positivity were significantly associated with a pCR. In terms of quantitative measurement values of breast MRI, the tumor–fat interface volume was significantly lower in patients with pCR than those without pCR (*p* = 0.003 and *p* = 0.004 for the development and validation cohorts, respectively).

### 3.2. Relationship between the Tumor-Fat Interface Volume and Clinicopathological Factors

The median tumor–fat interface volume in the development cohort was 1.80 cm^3^ (range, 0.05–17.95 cm^3^; interquartile range (IQR), 1.01–3.46 cm^3^). The optimal cutoff value calculated by the Youden’s J index in the development cohort was 2.36 cm^3^. Using this cutoff value, patients were divided into either the high interface group (tumor–fat interface volume ≥ 2.36 cm^3^) or the low interface group (tumor–fat interface volume < 2.36 cm^3^).

The characteristics of patients according to interface groups are shown in Table 2. Younger age (*p* < 0.001 and *p* = 0.008 for the development and validation cohorts, respectively) and larger tumor volume (*p* < 0.001 and *p* < 0.001 for the development and validation cohorts, respectively) were associated with the high interface group. Higher BPE was significantly associated with a higher interface group (*p* < 0.001 and *p* = 0.027 for the development and validation cohorts, respectively). Patients classified into the high interface group experienced a pCR less frequently than those classified into the low interface group (*p* < 0.001 and *p* = 0.008 for the development and validation cohorts, respectively).

### 3.3. Factors Associated with Pathologic Response in the Development Cohort

**Figure 3:**
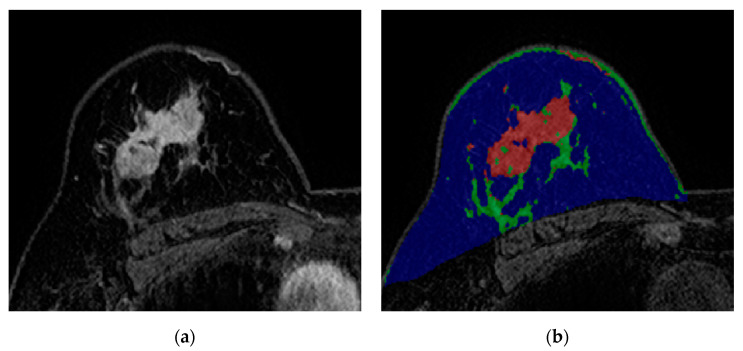
Images from breast MRI of a 59-year-old woman with invasive ductal carcinoma in the right breast. (**a**) Contrast-enhanced T1-weighted image showing a 4.4 cm malignant mass. This image depicts abundant peri-tumoral adipose tissue. (**b**) A color-overlay image showing differentiation between fat (blue), normal fibroglandular tissue (green), and tumor (red). In this patient, the tumor-fat interface volume was 4.33 cm^3^ and classified as high interface group. Final pathology after surgery revealed a non-pCR.

The results of univariable and multivariable logistic regression analysis are shown in Table 3. Multivariable analysis indicated that higher T stage (T3 stage, odds ratio (OR) = 2.122 (95% CI = 1.046–4.306), *p* = 0.037; T4 stage, OR = 5.655 (95% CI = 1.903–16.808), *p* = 0.002), higher N stage (N3 stage, OR = 2.237 (95% CI = 1.304–3.837), *p* = 0.003), HER2-negativity (OR = 5.002 (95% CI = 3.691–6.777), *p* < 0.001), and high interface group (OR = 1.412 (95% CI = 1.033–1.929), *p* = 0.030) remained independent factors for a non-pCR (Figure 3; Table 3). The multivariable analysis excluding the interface group is shown in Supplementary Table S1.

### 3.4. Validation of the Tumor-Fat Interface Volume in the Validation Cohort and Comparison of Model Performance

Development and validation cohort patient characteristics are shown in Table S2. Tumor volume (cm^3^), tumor–fat interface volume (cm^3^), and pCR rates were not significantly different between the two cohorts. Table 4 shows univariable and multivariable analyses of the validation cohort. Univariable analysis indicated that the high interface group was less likely to have a pCR with statistical significance (*p* = 0.009) (Table 4). Multivariable logistic regression analysis indicated that HER2-negativity (OR = 3.481 (95% CI = 1.482–8.173), *p* = 0.004), lower Ki-67 (OR = 5.463 (95% CI = 0.997–29.948), *p* = 0.050), and the high interface group (OR = 3.488 (95% CI = 1.403–8.675), *p* = 0.007) remained independent factors of a non-pCR (Table 4).

In the development cohort, the area under the curve (AUC) value of the conventional prediction model (Supplementary Table S1) was 0.767 (95% CI = 0.738–0.797) while the combined model was 0.770 (95% CI = 0.740–0.799). The *p*-value for the DeLong test was 0.767. The NRI and IDI were −0.005 (95% CI = −0.032–0.023; *p* = 0.746) and 0.004 (95% CI = 0.0001–0.008; *p* = 0.047), respectively. AIC values for the conventional prediction model and the combined model were 1125.934 and 1123.214, respectively. In the validation cohort, the AUC value of the conventional prediction model was 0.796 (95% CI = 0.723–0.869) while the combined prediction. The results of Spearman’s rank correlation are in Supplementary Figure S1.

Model was 0.830 (95% CI = 0.763–0.897). The *p*-value for the DeLong test was 0.29. NRI and IDI were 0.123 (95% CI = −0.067–0.313; *p* = 0.206) and 0.068 (95% CI = 0.023–0.113; *p* = 0.003), respectively. AIC values for the conventional prediction model and the combined model were 165.867 and 157.364, respectively. We next evaluated whether the prognostic role of the fat-tumor interface was different according to molecular subtype. In patients with HR−/HER2+ cancer (OR = 1.933, 95% CI = 1.037–3.604, *p* = 0.038), high interface group was significantly associated with worse outcome (Supplementary Table S3).

The interobserver agreement of the quantitative measurement values between the two readers was extremely high. The mean intraclass correlation coefficient (ICC) for all measurement values was 0.972. The ICC value was 0.986 for breast volume, 0.990 for fat volume, 0.954 for normal fibroglandular tissue volume, 0.985 for tumor volume, and 0.943 for tumor–fat interface volume.

## 4. Discussion

Our results indicate that a high tumor–fat interface volume was an independent risk factor for predicting a non-pCR. We confirmed our results in an external validation cohort. In our results, the tumor–fat interface volume does not seem to correlate with other prognostic factors such as BMI, ER, PR, or HER2. Younger age, higher BPE, and larger tumor volume were significantly associated with the high interface group in both the development and validation cohorts. We assume that volume of direct contact with the neighboring fat increases as the tumor grows. With regards to younger age, breast cancer in young women is known to be larger and have more multifocality than that in older women [22,23] and this could increase the tumor–fat interface volume in younger women. Young age is known to be associated with high BPE and this could explain why higher BPE was significantly related to the high interface group. Our results indicate that younger women have a larger tumor–fat interface volume, and this increased volume could actively work as an endocrine organ, especially with regards to functioning in a paracrine manner. We hypothesize that this might partially explain why breast cancer in young women usually has worse outcomes. In our study, BMI did not have significant predictive value with regards to NAC response in both the development cohort and validation cohort. BMI is easily measurable and has been most commonly used in this research field. However, the inherent inadequacy of the parameter may account for some of conflicting epidemiological data that argues for and against positive associations between BMI and cancer progression [24,25].

Although extensive research has been conducted regarding the biological mechanisms between adipose tissue and cancer, few studies have evaluated the impact of the local fat environment in breast cancer patients using imaging modalities. Stacy-Clear et al. reported that most cancers (63 of 86) detected by mammography were peripherally located within a 1-cm wide zone beneath the subcutaneous or retromammary fat [26]. However, they concluded that lesion location was secondary to breast geometry, with the peripheral zone simply accounting for a larger breast volume. Kim et al. evaluated the location of malignant and benign breast lesions with regard to the fat–gland interface using MRI [27]. They found that malignant lesions and more invasive cancers tended to be located in or near the fat–gland interface compared with benign lesions or ductal carcinoma in situ (DCIS). Their results showed that a greater proportion of malignant lesions were located in or near the fat-gland interface through qualitative and quantitative analysis. They insisted that the fat-gland interface location of breast cancer is not due to chance, but instead reflects a biological phenomenon. More recently, Obeid et al. proposed the peri-tumoral fat ratio to predict axillary nodal metastasis in breast cancer patients using breast MRI [28]. They segmented the tumor and then generated 1 cm peri-tumoral shell expansions. In their study, a categorical combination of BMI with higher peri-tumoral fat ratio yielded the strongest correlation regarding nodal metastasis in obese patients.

Compared with prior studies that focused on the relationship between fat in the breast and tumor, our study has several notable merits. First, quantitative measurement of the tumor–fat interface volume rather than using shells surrounding the tumors [28,29] was attempted for the first time. Two prior papers adopted an approach to expand the tumor region of interest to calculate the tumor–fat ratio [28,29]. This approach is problematic as the amount of expansion in the form of a shell is arbitrary and would lead to variability in the tumor–fat ratio. Adipocytes neighboring breast cancer surely affect tumor progression and response to therapy not only in the contact plane with the tumor but also in regions further away from the plane. However, it is impossible to define the spatial extents of the adipocytes to include. Therefore, we just measured the direct contact plane. Second, contrary to prior studies, there was no constraint on inclusion of patients with multiple masses or non-mass enhancement type tumors in our study. Therefore, our study has wider implications. Third, our methodology is objective and easy to follow. This semi-automatic method allows more accurate calculations of the tumor–fat interface volume. Intra- or inter-observer agreement should be high for this kind of technology to be widely used. The ICC value between the two tumor–fat interface volume measurements made by different radiologists was 0.943, indicating excellent interobserver agreement. Fourth, our results were derived from a large number of patients, and thus are statistically robust. Moreover, we confirmed our results using an external cohort.

However, there are still several limitations in this study. First, there was no ground truth to determine tumor extent. As all patients underwent NAC, the disease extent as assessed by pretreatment MRI would be different from the actual extent of the pathologic disease. However, this is an inevitable limitation of this type of research. Second, we used the k-means clustering technique to calculate the tumor–fat interface. In some cases, mimics such as blood vessels or strong background parenchymal enhancement (BPE) affected measurements. However, this was insignificant when considering whole tumor volume. Nevertheless, we cannot fully ignore the effects of BPE especially when it shows similar degree of enhancement with breast cancer. In addition, we excluded patients who had ambiguous tumor extents largely caused by high BPE. Therefore, our method might have to be used with caution in patients with high BPE. Third, although we showed that the tumor–fat interface volume significantly affected the treatment response to NAC, the biological mechanisms of this phenomenon remain unclear. We believe this is mainly due to the paracrine effect of adipose tissue. However, further biochemical studies are required in order to explain how this works. Fourth, we used the tumor–fat interface volume itself without adjusting it for possible covariates such as tumor volume or BMI. Fifth, there were differences in adopted contrast agents and chemotherapy regimens between two cohorts. Our study is a retrospective one and thus matching the setting between cohorts was not practical. Our results should be interpreted with potential confounds from multi-center differences in mind. Future prospective studies with matched settings are needed to fully validate our results. Sixth, our method could not be applied to all cases. Some cases (i.e., 24 cases) could not be delineated due to the ambiguous tumor extents. Better imaging acquisition combined with enhanced clustering might solve this issue. Still, this needs to be further developed and tested in future studies. Finally, this was a retrospective study. To fully confirm the prognostic value of tumor–fat interface volume as a new imaging biomarker, prospective multi-institutional studies are needed.

## 5. Conclusions

In conclusion, our results suggest that the tumor–fat interface volume could have the potential to be used as a prognostic imaging biomarker to predict treatment response to NAC.

## Figures and Tables

**Table 1:** Characteristics of patients.

Characteristics	Development Cohort			Validation Cohort		
	pCR (*n* = 369)	Non-pCR (*n* = 635)	*p*-Value	pCR (*n* = 45)	Non-pCR (*n* = 91)	*p*-Value
Age (median [IQR])	49.00 [41.00, 56.00]	47.00 [40.00, 54.00]	0.028	50.00 [45.00, 58.00]	52.00 [46.00, 57.50]	0.985
Breast volume (cm^3^) (median [IQR])	560.96 [388.82, 770.62]	580.17 [395.30, 805.52]	0.548	618.33 [427.09, 811.17]	675.34 [430.01, 944.99]	0.200
Fat volume (cm^3^) (median [IQR])	415.50 [250.97, 603.07]	407.79 [263.06, 623.10]	0.859	467.77 [308.59, 567.21]	508.43 [322.12, 749.08]	0.289
Normal fibroglandular tissue volume (cm^3^) (median [IQR])	125.32 [88.26, 177.29]	126.58 [88.27, 178.84]	0.792	115.51 [81.39, 186.44]	142.64 [87.27, 200.07]	0.420
Tumor volume (cm^3^) (median [IQR])	9.93 [6.05, 18.21]	13.45 [7.54, 24.81]	<0.001	9.67 [6.75, 19.48]	14.62 [6.65, 22.13]	0.184
Tumor-fat interface volume (cm^3^) (median [IQR])	1.64 [0.86, 2.85]	1.93 [1.08, 3.66]	0.003	1.36 [0.83, 2.21]	2.26 [1.33, 3.66]	0.004
Operation method			<0.001			>0.999
Breast-conserving surgery	298 (80.8)	361 (56.9)		33 (73.3)	68 (74.7)	
Mastectomy	71 (19.2)	274 (43.1)		12 (26.7)	23 (25.3)	
BMI	23.60 [21.80, 25.83]	23.63 [21.67, 26.13]	0.817	25.22 [22.43, 27.73]	24.95 [23.18, 27.76]	0.627
BMI			0.775			>0.999
<25 (kg/m^2^)	240 (65.0)	420 (66.1)		22 (48.9)	46 (50.5)	
≥25 (kg/m^2^)	129 (35.0)	215 (33.9)		23 (51.1)	45 (49.5)	
NAC regimen			<0.001			0.004
AC-T	133 (36.0)	443 (69.8)		17 (37.8)	56 (61.5)	
AC-T/Herceptin	111 (30.1)	132 (20.8)		9 (20.0)	16 (17.6)	
TCHP	122 (33.1)	55 (8.7)		19 (42.2)	15 (16.5)	
AC	3 (0.8)	5 (0.8)		0 (0.0)	4 (4.4)	
cT stage at diagnosis			<0.001			0.671
1	20 (5.4)	26 (4.1)		2 (4.4)	3 (3.3)	
2	260 (70.5)	381 (60.0)		33 (73.3)	59 (64.8)	
3	82 (22.2)	181 (28.5)		9 (20.0)	24 (26.4)	
4	7 (1.9)	47 (7.4)		1 (2.2)	5 (5.5)	
cN stage at diagnosis			<0.001			0.901
0	58 (15.7)	58 (9.1)		4 (8.9)	6 (6.6)	
1	131 (35.5)	193 (30.4)		31 (68.9)	60 (65.9)	
2	129 (35.0)	228 (35.9)		4 (8.9)	10 (11.0)	
3	51 (13.8)	156 (24.6)		6 (13.3)	15 (16.5)	
Estrogen receptor			<0.001			<0.001
Positive	122 (33.1)	329 (51.8)		16 (35.6)	64 (70.3)	
Negative	247 (66.9)	306 (48.2)		29 (64.4)	27 (29.7)	
Progesterone receptor			<0.001			<0.001
Positive	61 (16.5)	258 (40.6)		12 (26.7)	55 (60.4)	
Negative	308 (83.5)	377 (59.4)		33 (73.3)	36 (39.6)	
HER2			<0.001			0.003
Positive	233 (63.1)	187 (29.4)		28 (62.2)	31 (34.1)	
Negative	136 (36.9)	448 (70.6)		17 (37.8)	60 (65.9)	
Ki-67			0.007			0.012
≥20%	336 (91.1)	539 (84.9)		43 (95.6)	72 (79.1)	
<20%	33 (8.9)	96 (15.1)		2 (4.4)	19 (20.9)	
Molecular subtype			<0.001			<0.001
HR+/HER2−	38 (10.3)	236 (37.2)		5 (11.1)	45 (49.5)	
HR+/HER2+	89 (24.1)	105 (16.5)		13 (28.9)	20 (22.0)	
HR−/HER2+	144 (39.0)	82 (12.9)		15 (33.3)	11 (12.1)	
HR−/HER2−	98 (26.6)	212 (33.4)		12 (26.7)	15 (16.5)	
Menopausal status			0.027			>0.999
Postmenopausal	170 (46.1)	246 (38.7)		24 (53.3)	48 (52.7)	
Premenopausal	199 (53.9)	389 (61.3)		21 (46.7)	43 (47.3)	
Background parenchymal enhancement (BPE)			0.579			0.465
1	166(45.0)	290(45.7)		27 (60.0)	43 (47.3)	
2	102 (27.6)	156 (24.6)		11 (24.4)	26 (28.6)	
3	52 (14.1)	88 (13.9)		6 (13.3)	15 (16.5)	
4	49 (13.3)	101 (15.9)		1 (2.2)	7 (7.7)	
Mammographic breast density			0.788			0.695
1	7 (1.9)	11 (1.7)		0 (0.0)	1 (1.1)	
2	57 (15.4)	114 (18.0)		7 (15.6)	13 (14.3)	
3	183 (49.6)	305 (48.0)		18 (40.0)	45 (49.5)	
4	122 (33.1)	205 (32.3)		20 (44.4)	32 (35.2)	

NOTE: Unless otherwise noted, data indicate numbers of patients with percentages in parentheses. IQR, interquartile range; BMI, body mass index.

**Table 2:** Characteristics of patients according to optimal cutoff for tumor-fat interface volume in the development and validation cohorts.

Characteristics	Development Cohort			Validation Cohort		
	Low (*n* = 608)	High (*n* = 396)	*p*-Value	Low (*n* = 81)	High (*n* = 55)	*p*-Value
Age (median [IQR])	49.00 [42.00, 56.00]	45.00 [38.00, 53.00]	<0.001	53.00 [48.00, 59.00]	48.00 [43.00, 55.00]	0.008
Breast volume (cm^3^) (median [IQR])	509.50 [353.82, 717.03]	664.70 [455.60, 904.62]	<0.001	628.36 [426.52, 806.93]	684.99 [505.84, 1032.36]	0.073
Fat volume (cm^3^) (median [IQR])	384.16 [230.58, 550.14]	477.44 [288.45, 687.47]	<0.001	455.51 [319.60, 593.58]	535.64 [295.53, 822.07]	0.178
Normal fibroglandular tissue volume (cm^3^) (median [IQR])	115.10 [80.72, 158.67]	147.88 [103.89, 202.52]	<0.001	121.35 [82.63, 164.36]	152.97 [89.65, 227.67]	0.063
Tumor volume (cm^3^) (median [IQR])	8.01 [5.35, 12.07]	23.82 [16.15, 35.74]	<0.001	7.08 [5.32, 11.64]	22.04 [15.80, 32.68]	<0.001
Operation method						
Breast-conserving surgery	442 (72.7)	217 (54.8)	<0.001	65 (80.3)	36 (65.5)	0.053
Mastectomy	166 (27.3)	179 (45.2)		16 (19.7)	19 (34.5)	
BMI (median [IQR])	23.47 [21.61, 25.65]	23.99 [21.82, 26.64]	0.006	24.87 [22.49 26.73]	25.20 [23.21 28.65]	0.188
BMI						
<25 (kg/m^2^)	420 (69.1)	240 (60.6)	0.007	42 (51.8)	26 (47.3)	0.600
≥25 (kg/m^2^)	188 (30.9)	156 (39.4)		39 (48.2)	29 (52.7)	
NAC regimen						
AC-T	334 (54.9)	242 (61.1)	0.103	45 (55.5)	28 (50.9)	0.519
AC-T/Herceptin	157 (25.8)	86 (21.7)		17 (21.0)	8 (45.6)	
TCHP	114 (18.8)	63 (15.9)		17 (21.0)	17 (30.9)	
AC	3 (0.5)	5 (1.3)		2 (2.5)	2 (3.6)	
cT stage at diagnosis						
1	27 (4.4)	19 (4.8)	<0.001	3 (3.7)	2 (3.6)	0.008
2	446 (73.4)	195 (49.2)		63 (77.8)	29 (52.7)	
3	122 (20.1)	141 (35.6)		12 (14.8)	21 (38.2)	
4	13 (2.1)	41 (10.4)		3 (3.7)	3 (5.5)	
cN stage at diagnosis						
0	76 (12.5)	40 (10.1)	0.107	7 (8.6)	3 (5.5)	0.633
1	206 (33.9)	118 (29.8)		55 (67.9)	36 (65.4)	
2	214 (35.2)	143 (36.1)		9 (11.1)	5 (9.1)	
3	112 (18.4)	95 (24.0)		10 (12.4)	11 (20.0)	
Estrogen receptor						
Positive	273 (44.9)	178 (44.9)	>0.999	43 (53.1)	37 (67.3)	0.099
Negative	335 (55.1)	218 (55.1)		38 (46.9)	18 (32.7)	
Progesterone receptor						
Positive	191 (31.4)	128 (32.3)	0.816	34 (42.0)	33 (60.0)	0.039
Negative	417 (68.6)	268 (67.7)		47 (58.0)	22 (40.0)	
HER2						
Positive	271 (44.6)	149 (37.6)	0.034	34 (42.0)	25 (45.4)	0.688
Negative	337 (55.4)	247 (62.4)		47 (58.0)	30 (54.6)	
Ki-67						
≥20%	524 (86.2)	351 (88.6)	0.299	66 (81.5)	49 (89.1)	0.228
<20%	84 (13.8)	45 (11.4)		15 (18.5)	6 (10.9)	
Molecular subtype						
HR+/HER2−	154 (25.3)	120 (30.3)	0.12	27 (33.3)	23 (41.8)	0.335
HR+/HER2+	129 (21.2)	65 (16.4)		18 (22.2)	15 (27.3)	
HR−/HER2+	142 (23.4)	84 (21.2)		16 (19.8)	10 (18.2)	
HR−/HER2−	183 (30.1)	127 (32.1)		20 (24.7)	7 (12.7)	
Menopausal status						
Postmenopausal	278 (45.7)	138 (34.8)	0.001	48 (59.3)	24 (43.6)	0.073
Premenopausal	330 (54.3)	258 (65.2)		33 (40.7)	31 (56.4)	
Background parenchymal enhancement (BPE)						
1	308 (50.7)	148 (37.4)	<0.001	47 (58.0)	23 (41.8)	0.027
2	154 (25.3)	104 (26.3)		21 (25.9)	16 (29.1)	
3	76 (12.5)	64 (16.2)		12 (14.8)	9 (16.4)	
4	70 (11.5)	80 (20.2)		1 (1.2)	7 (12.7)	
Mammographic breast density						
1	10 (1.6)	8 (2.0)	0.439	2 (2.5)	2 (3.6)	0.960
2	108 (17.8)	63 (15.9)		12 (14.8)	8 (14.6)	
3	303 (49.8)	185 (46.7)		37 (45.7)	23 (41.8)	
4	187 (30.8)	140 (35.4)		30 (37.0)	22 (40.0)	
Pathologic response						
pCR	250 (41.1)	119 (30.1)	<0.001	34 (42.0)	11 (20.0)	0.008
Non-pCR	358 (58.9)	277 (69.9)		47 (58.0)	44 (80.0)	

NOTE: Unless otherwise noted, data indicate number of patients with percentages in parentheses. IQR, interquartile range; BMI, body mass index.

**Table 3:** Univariable and multivariable logistic regression analyses of factors associated with non-pCR in the development cohort.

Variable	Univariable			Multivariable		
	Odds Ratio	95% CI	*p*-Value	Odds Ratio	95% CI	*p*-Value
Age	0.986	0.973, 0.999	0.036	1.009	0.985, 1.033	0.478
Breast volume (cm^3^)	1.0002	0.9996, 1.0008	0.456			
Fat volume (cm^3^)	1.0003	0.9995, 1.0010	0.719			
Normal fibroglandular tissue volume (cm^3^)	1.0000	0.9979, 1.0020	0.496			
Tumor volume (cm^3^)	1.0098	0.9990, 1.0206	<0.001			
BMI						
<25 (kg/m^2^)	Ref.			Ref		
≥25 (kg/m^2^)	0.952	0.727, 1.247	0.723	0.925	0.679, 1.258	0.618
NAC regimen			<0.001			
AC-T	Ref.					
AC-T/Herceptin	0.357	0.260, 0.491	<0.001			
TCHP	0.135	0.093, 0.196	<0.001			
AC	0.500	0.118, 2.121	0.347			
cT stage at diagnosis			<0.001			
1	Ref			Ref		
2	1.127	0.616, 2.062	0.698	1.472	0.758, 2.856	0.253
3	1.698	0.896, 3.216	0.104	2.122	1.046, 4.306	0.037
4	5.165	1.929, 13.831	0.001	5.655	1.903, 16.808	0.002
cN stage at diagnosis			<0.001			
0	Ref			Ref		
1	1.473	0.962, 2.256	0.075	1.271	0.793, 2.038	0.318
2	1.767	1.158, 2.699	0.008	1.555	0.973, 2.485	0.065
3	3.059	1.889, 4.954	<0.001	2.237	1.304, 3.837	0.003
Estrogen receptor						
Positive	Ref			Ref		
Negative	0.459	0.352, 0.600	<0.001	0.892	0.606, 1.313	0.562
Progesterone receptor						
Positive	Ref			Ref		
Negative	0.289	0.211, 0.397	<0.001	0.268	0.169, 0.425	<0.001
HER2						
Positive	Ref			Ref		
Negative	4.104	3.128, 5.386	<0.001	5.002	3.691, 6.777	<0.001
Ki-67						
≥20%	Ref			Ref		
<20%	1.813	1.194, 2.755	0.005	1.521	0.932, 2.482	0.093
Molecular subtype			<0.001			
HR+/HER2−	Ref		<0.001			
HR+/HER2+	0.190	0.122, 0.296	<0.001			
HR−/HER2+	0.092	0.059, 0.142	<0.001			
HR−/HER2−	0.348	0.229, 0.529	<0.001			
Menopausal status						
Postmenopausal	Ref			Ref		
Premenopausal	1.351	1.042, 1.751	0.023	0.889	0.561, 1.409	0.617
Background parenchymal enhancement (BPE)			0.580			
1	Ref.					
2	0.875	0.639, 1.199	0.407			
3	0.969	0.654, 1.434	0.874			
4	1.180	0.798, 1.744	0.407			
Mammographic breast density			0.788			
1	Ref					
2	1.273	0.468, 3.458	0.636			
3	1.061	0.404, 2.784	0.905			
4	1.069	0.404, 2.831	0.893			
Tumor-fat interface volume						
Low	Ref			Ref		
High	1.626	1.242, 2.127	<0.001	1.412	1.033, 1.929	0.030

**Table 4:** Univariable and multivariable logistic regression analyses of factors associated with non-pCR in the validation cohort.

Variable	Univariable			Multivariable		
	Odds Ratio	95% CI	*p*-Value	Odds Ratio	95% CI	*p*-Value
Age	1.0004	0.958, 1.044	0.985			
Breast volume (cm^3^)	1.003	1.001, 1.005	0.154			
Fat volume (cm^3^)	1.003	1.000, 1.005	0.139			
Normal fibroglandular tissue volume (cm^3^)	1.004	0.998, 1.109	0.731			
Tumor volume (cm^3^)	1.055	1.003, 1.109	0.104			
BMI						
<25 (kg/m^2^)	Ref					
≥25 (kg/m^2^)	0.936	0.458, 1.912	0.855			
NAC regimen			0.015			
AC-T	Ref.					
AC-T/Herceptin	0.540	0.202, 1.439	0.218			
TCHP	0.240	0.101, 0.571	0.001			
AC	>999.999	<0.001, >999.999	0.990			
cT stage at diagnosis			0.658			
1	Ref					
2	1.192	0.189, 7.499	0.852			
3	1.778	0.254, 12.450	0.562			
4	3.333	0.204, 54.535	0.398			
cN stage at diagnosis			0.902			
0	Ref					
1	1.290	0.339, 4.915	0.709			
2	1.667	0.300, 9.272	0.560			
3	1.667	0.343, 8.093	0.526			
Estrogen receptor						
Positive	Ref			Ref		
Negative	0.233	0.109, 0.497	<0.001	0.302	0.092, 0.995	0.049
Progesterone receptor						
Positive	Ref			Ref		
Negative	0.238	0.109, 0.521	<0.001	0.838	0.245, 2.870	0.778
HER2						
Positive	Ref			Ref		
Negative	3.188	1.517, 6.697	0.002	3.481	1.482, 8.173	0.004
Ki-67						
≥20%	Ref			Ref		
<20%	5.674	1.260, 25.556	0.024	5.463	0.997, 29.948	0.050
Molecular subtype			0.001			
HR+/HER2−	Ref					
HR+/HER2+	0.171	0.054, 0.544	0.003			
HR−/HER2+	0.081	0.024, 0.273	<0.001			
HR−/HER2−	0.139	0.042, 0.459	0.001			
Menopausal status						
Postmenopausal	Ref					
Premenopausal	1.024	0.500, 2.095	0.949			
Background parenchymal enhancement (BPE)			0.448			
1	Ref.					
2	1.484	0.632, 3.485	0.365			
3	1.570	0.543, 4.540	0.405			
4	4.395	0.512, 37.729	0.177			
Mammographic breast density			0.735			
1	Ref					
2	<0.001	<0.001, >999.999	0.987			
3	<0.001	<0.001, >999.999	0.988			
4	<0.001	<0.001, >999.999	0.987			
Tumor-fat interface volume						
Low	Ref			Ref		
High	2.894	1.307, 6.405	0.009	3.488	1.403, 8.675	0.007

## Supplementary Materials

The following are available online at https://www.mdpi.com/2079-7737/9/11/391/s1, Table S1: Multivariable logistic regression model for predicting non-pCR excluding the tumor-fat interface volume, Table S2: Characteristics of patients in the development set and validation set, Table S3: Summarized result of the subgroup analysis for the significance of high tumor-fat interface volume group according to molecular subtypes in the development cohort.
