# Supplementary material for: The Tumor–Fat Interface Volume of Breast Cancer on Pretreatment MRI Is Associated with a Pathologic Response to Neoadjuvant Chemotherapy
Source: Biology (Basel). 2020 Nov 10;9(11):391. doi: 10.3390/biology9110391 (PMC7697338; doi:10.3390/biology9110391)
Supplement: Supplementary file 1 [file biology-09-00391-s001.pdf]

S1. MRI protocol

The following describes the data acquisition procedures at different hospitals. For the SMC cohort, all MRI scans were performed on either a 1.5 T or 3.0 T scanners both from Philips (1.5 T Achieva and 3.0 T Achieva, Philips Healthcare, Best, The Netherlands). The MRI examination consisted of turbo spin-echo T1- and T2-weighted sequences and a fat-suppressed 3-dimensional dynamic contrast-enhanced (DCE) sequence. Image subtraction was performed after the dynamic series. The DCE-MRI scans were acquired on a .5 T scanner using the following parameters: TR/TE, 6.5/2.5; slice thickness, 1.5 mm; flip angle, 10°; matrix size, 376 × 374; and field of view, 32 × 32 cm. DCE-MRI was performed with axial imaging with 1 pre-contrast and 6 post-contrast dynamic series. Contrast-enhanced images were acquired at 30, 90, 150, 210, 270, and 330 s after contrast injection. The DCE timing was the center of the k-space acquisition, and the length of each dynamic series was 1.05 min. The DCE-MRI scans were acquired on a 3.0 T scanner using the following parameters: TR/TE, 5.5/2.8; slice thickness, 3 mm; flip angle, 12°; matrix size, 500 × 237; and field of view, 30 x 30 cm. Fat-suppressed DCE-MRI was performed with axial imaging, with 1 pre-contrast and 6 post-contrast dynamic series. Contrast-enhanced images were acquired at 30, 90, 150, 210, 270, and 330 s after contrast injection. For dynamic contrast enhancement, a 0.1 mmol/kg bolus of gadobutrol (Gadovist; Bayer Healthcare Pharmaceutical, Berlin, Germany) was injected, followed by a 20ml saline flush.

For the NCC cohort, all MRI scans were performed on a 3.0 T Philips scanner (Achieva, Philips Healthcare, Best, The Netherlands). The MRI examination consisted of turbo spin-echo T1- and T2-weighted sequences and a fat-suppressed 3-dimensional DCE sequence. Image subtraction was performed after the dynamic series. The DCE-MRI scans were acquired using the following parameters: TR/TE, 5.5/2.8; slice thickness, 3 mm; flip angle,

18°; matrix size, 424 x 424; and field of view, 34 x 34 cm. DCE-MRI was performed with axial imaging, with 1 pre-contrast and 5 post-contrast dynamic series. Contrast-enhanced axial images were acquired at 1.5, 3, 4.5, and 6 min after contrast injection. A delayed sagittal image was obtained 8 min after contrast injection. Image subtraction was performed after the dynamic series. For dynamic contrast enhancement, a 0.1 mmol/kg bolus of gadoterate meglumine (Dotarem; Guerbet and Clariscan, GE Healthcare, USA) was injected, followed by a 20 ml saline flush.

S2. Technical detail

The manually delineated chest walls were interpolated using B-splines to fully cover the extent of the breast. To segment the breast region, a search region was set up at the anterior portion of the chest wall. The pre-contrast T1-weighted image was smoothed using a Gaussian kernel with sigma of 2.5 mm for each slice to reduce noise. A k-means clustering algorithm with k=3 (background, breast, and noise regions) was performed for the search region, and we chose the cluster with the highest intensity as the candidate breast region. This was because the breast region appeared brighter than other regions on the pre-contrast T1-weighted image. The results were further refined with a median filter, hole filling, and morphological erosion with sigma 2 times that used in Gaussian smoothing. Erosion was applied to adjust for the blurring effect induced by Gaussian smoothing. The refined result was considered as the final segmented breast. The segmented breast defined on pre-contrast images were transferred to various post-contrast images. To separate the fat and non-fat regions, a k-means clustering algorithm with k=2 (fat vs. non-fat) was applied to pre-contrast images for each slice within the segmented breast. This was because the fat region appeared darker on fat-suppressed pre-contrast images. The non-fat region was further subdivided using another k-means clustering with k=3 (normal parenchyma, tumor, and others) using

concatenated subtraction images (90 s minus pre-contrast) and a delayed phase (at 330 s for SMC and 360 s for NCC after contract injection) for each slice.The results were further refined with median filter and morphological operations leading to the final segmented breast.The tumor region was determined as the cluster showing the most rapid and strongest enhancement among the three clusters.

TableS1. Multivariable logistic regression model for predicting non-pCR excluding the tumor-fat interface volume.

Variable	Non-pCR		*p*-value
	Odds ratio	95% CI	
Age	1.006	0.983, 1.030	0.618
BMI			
<25 kg/m^2	Ref		
≥ 25 kg/m^2	0.949	0.699, 1.290	0.739
cT stage at diagnosis			
1	Ref		
2	1.416	0.732, 2.736	0.301
3	2.190	1.083, 4.428	0.029
4	6.207	2.111, 18.247	0.001
cN stage at diagnosis			
0	Ref		
1	1.282	0.801, 2.053	0.301
2	1.563	0.979, 2.496	0.061
3	2.260	1.319, 3.872	0.003
Estrogen receptor			
Positive	Ref		
Negative	0.899	0.612, 1.321	0.588
Progesterone receptor			
Positive	Ref		
Negative	0.270	0.170, 0.427	<0.001
HER2			
Positive	Ref		
Negative	5.069	3.743, 6.865	<0.001
Ki-67			
≥20%	Ref		
<20%	1.490	0.915, 2.428	0.109
Menopausal status			
Postmenopausal	Ref		
Premenopausal	0.889	0.562, 1.407	0.616

TableS2 Characteristics of patients in the development set and validation set.

Characteristics	SMC cohort (*n* = 1004)	NCC cohort (*n* = 136)	*p*-value
Age (median [IQR])	48.00 [40.00, 54.00]	52.00 [46.00, 58.00]	<0.001
Breast volume (cm^3) (median [IQR])	569.39 [391.72, 787.91]	647.96 [428.11, 911.63]	0.013
Fat volume (cm^3) (median [IQR])	408.24 [256.91, 621.73]	492.85 [316.40, 705.93]	0.013
Normal fibroglandular tissue volume (cm^3) (median [IQR])	126.42 [88.25, 178.84]	134.69 [84.62, 193.72]	0.438
Tumor volume (cm^3) (median [IQR])	12.13 [6.85, 21.64]	11.78 [6.68, 21.93]	0.997
Tumor-fat interface volume (cm^3) (median [IQR])	1.80 [1.01, 3.46]	1.92 [1.02, 3.16]	0.760
Operation method			0.057
Breast-conserving surgery	659 (65.6)	101 (74.3)	
Mastectomy	345 (34.4)	35 (25.7)	
BMI (median [IQR])	23.62 [21.73, 25.99]	24.98 [22.59, 27.74]	<0.001
BMI			0.001
<25 kg/m^2	659 (65.6)	68 (50.0)	
≥ 25 kg/m^2	345 (34.4)	68 (50.0)	
NAC regimen			
AC-D	576 (57.4)	73 (53.7)	0.016
AC-D/Herceptin	243 (24.2)	25 (18.4)	
TCHP	177 (17.6)	34 (25.0)	
AC	8 (0.8)	4 (2.9)	
cT stage at diagnosis			0.905
1	46 (4.6)	5 (3.7)	
2	641 (63.8)	92 (67.6)	
3	263 (26.2)	33 (24.3)	
4	54 (5.4)	6 (4.4)	
cN stage at diagnosis			
0	116 (11.6)	10 (7.4)	<0.001
1	324 (32.3)	91 (66.9)	
2	357 (35.6)	14 (10.3)	
3	207 (20.6)	21 (15.4)	
Estrogen receptor			
Positive	451 (44.9)	80 (58.8)	0.003
Negative	553 (55.1)	56 (41.2)	
Progesterone receptor			
Positive	319 (31.8)	67 (49.3)	<0.001
Negative	685 (68.2)	69 (50.7)	
HER2			
Positive	420 (41.8)	59 (43.4)	0.802

Negative	584 (58.2)	77 (56.6)	
Ki-67			
≥20%	875 (87.2)	115 (84.6)	0.481
<20%	129 (12.8)	21 (15.4)	
Molecular subtype			0.013
$HR^+/HER2^-$	274 (27.3)	50 (36.8)	
$HR^+/HER2^+$	194 (19.3)	33 (24.3)	
$HR^-/HER2^+$	226 (22.5)	26 (19.1)	
HR-/HER2-	310 (30.9)	27 (19.9)	
Menopausal status			
Postmenopausal	416 (41.4)	72 (52.9)	0.014
Premenopausal	588 (58.6)	64 (47.1)	
Mammographic breast density			
1	18 (1.8)	1 (0.7)	0.564
2	171 (17.0)	20 (14.7)	
3	488 (48.6)	63 (46.3)	
4	327 (32.6)	52 (38.2)	
Tumor-fat interface volume			
Low	608 (60.6)	81 (59.6)	0.896
High	396 (39.4)	55 (40.4)	
Pathologic response			
pCR	369 (36.8)	45 (33.1)	0.46
Non-pCR	635 (63.2)	91 (66.9)	

Table S3. Summarized result of the subgroup analysis for the significance of high tumor-fat interface volume group according to molecular subtypes in the development cohort.

Molecular subtype	Non-pCR		
	OR	95% CI	*p*-value
HR+/HER2- (n = 274)	0.940	0.409, 2.161	0.885
HR+/HER2+ (n = 194)	1.143	0.544, 2.401	0.724
HR-/HER2+ (n = 226)	1.933	1.037, 3.604	0.038
HR-/HER2- (n = 310)	1.450	0.844, 2.490	0.179

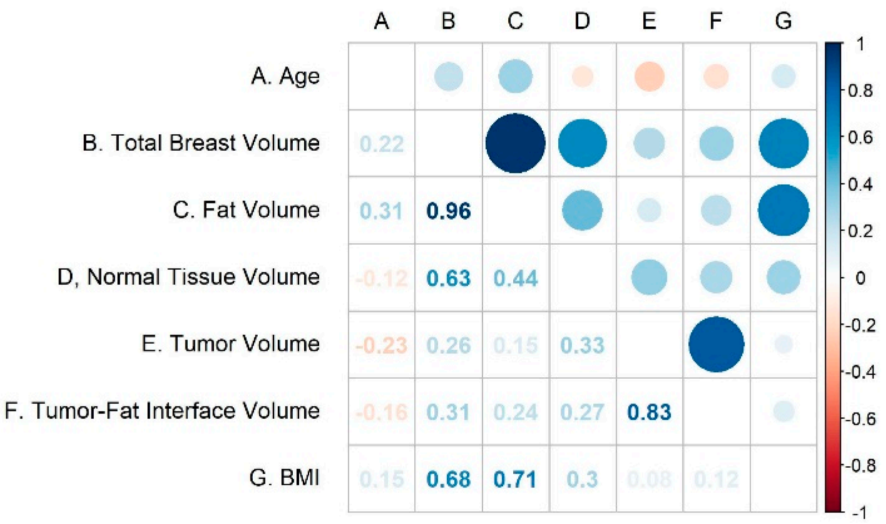

Figure S1. Size and color-coded matrix for Spearman's rank correlations between major measurements
